# Development of an artificial antibody specific for HLA/peptide complex derived from cancer stem-like cell/cancer-initiating cell antigen DNAJB8

**DOI:** 10.1038/s41416-020-1017-1

**Published:** 2020-08-05

**Authors:** Hiroki Tadano, Tomohide Tsukahara, Emi Mizushima, Asuka Akamatsu, Kazue Watanabe, Iyori Nojima, Terufumi Kubo, Takayuki Kanaseki, Yoshihiko Hirohashi, Noriyuki Sato, Toshihiko Torigoe

**Affiliations:** 1 Department of Pathology, Sapporo Medical University School of Medicine, South-1, West-17, Chuo-ku, Sapporo 060-8556 Japan; 2 Division of Internal Medicine, Sapporo Self-Defense Forces Hospital, 17-Makomanai, Minami-ku, Sapporo 005-8543 Japan; 3 Department of Orthopaedic Surgery, Sapporo Medical University School of Medicine, South-1, West-16, Chuo-ku, Sapporo 060-8543 Japan; 4 Department of Cancer Immunology, Medical and Biological Laboratories Co., Ltd, 1063-103, Terasawaoka, Ina 396-0002 Japan; 5 Division of Cell Bank, Biomedical Research, Education and Instrumentation Center, Sapporo Medical University School of Medicine, South-1, West-17, Chuo-ku, Sapporo 060-8556 Japan

**Keywords:** Immunoediting, Molecular medicine

## Abstract

**Background:**

Peptide-vaccination therapy targeting tumour-associated antigens can elicit immune responses, but cannot be used to eliminate large tumour burden. In this study, we developed a therapeutic single-chain variable-fragment (scFv) antibody that recognises the cancer stem-like cell/cancer-initiating cell (CSC/CIC) antigen, DNAJB8.

**Methods:**

We screened scFv clones reacting with HLA-A24:20/DNAJB8-derived peptide (DNAJB8_143) complex using naive scFv phage-display libraries. Reactivity and affinity of scFv clones against the cognate antigen were quantified using FACS and surface plasmon resonance. Candidate scFv clones were engineered to human IgG1 (hIgG1) and T-cell-engaging bispecific antibody (CD3xJB8). Complement-dependent cytotoxicity (CDC) and bispecific antibody-dependent cellular cytotoxicity (BADCC) were assessed.

**Results:**

scFv clones A10 and B10 were isolated after bio-panning. Both A10-hIgG1 and B10-hIgG1 reacted with DNAJB8-143 peptide-pulsed antigen-presenting cells and HLA-A24(+)/DNAJB8(+) renal cell carcinoma and osteosarcoma cell lines. A10-hIgG1 and B10-hIgG1 showed strong affinity with the cognate HLA/peptide complex (*K*_D_ = 2.96 × 10^−9^ M and 5.04 × 10^−9^ M, respectively). A10-hIgG1 and B10-hIgG1 showed CDC against HLA-A24(+)/DNAJB8(+) cell lines. B10-(CD3xJB8) showed superior BADCC to A10-(CD3xJB8).

**Conclusion:**

We isolated artificial scFv antibodies reactive to CSC/CIC antigen DNAJB8-derived peptide naturally present on renal cell carcinoma and sarcoma. Immunotherapy using these engineered antibodies could be promising.

## Background

The first immunotherapy against cancer was performed using Coley’s Toxins developed by William B. Coley in the nineteenth century.^1^ He vaccinated sarcoma patients with live or inactivated bacteria. Various vaccination therapies using tumour-associated antigen (TAA)-derived peptides, autologous tumour lysates and tumour lysate-loaded dendritic cells have been developed to date.^2^ We conducted peptide-vaccination trials using TAA-derived peptides of SYT–SSX junctional peptides derived from specific chromosomal translocation for synovial sarcoma, papillomavirus-binding factor (PBF) for osteosarcoma and survivin for various carcinomas.^3,4^ Although immune responses against the vaccinated peptides were frequently observed, the objective clinical response rates were low.

In contrast, cell-based immunotherapy using T cells exogenously expressing T-cell receptor (TCR) directed to TAA (TCR-T), especially for NY-ESO-I, showed clinical responses against melanoma and synovial sarcoma.^5^ Anti-CD19 chimeric antigen receptor-expressing T cells (CAR-T) also showed dramatic responses in B-cell haematological malignancies.^6^ However, CD19 CAR-T cells can destroy not only neoplastic B cells but also normal B cells. Target antigens are still restricted, and new antigens might be required for the development of immunotherapy for various cancers.

Considering the current status, we focused on (i) the identification of new TAAs that were expressed in cancer stem-like cells/cancer-initiating cells (CSCs/CICs) but not in normal organs that also regulate tumour-initiating ability, and (ii) the development of antibodies directed to CSC/CIC antigen using antibody engineering.^3,7^ Recently, various therapeutic antibodies targeting tumour antigens expressed on tumour cell surfaces have been developed. In particular, anti-CD20 monoclonal antibody rituximab and anti-CD19 T-cell-engaging bispecific antibody blinatumomab showed objective responses in B-cell haematological neoplasia.^8,9^

In this study, we developed a therapeutic single-chain variable-fragment (scFv) antibody recognising the HLA-A24/peptide derived from DNAJB8 complex as a prototype of CSC/CIC antigen.^10,11^ Then, we engineered the scFv to bivalent and bispecific formats to assess its in vitro cytotoxic capacity in renal cell carcinoma and sarcoma.

## Methods

This study was performed in accordance with the guidelines established by the Declaration of Helsinki, and was approved by the Ethics Committee of Sapporo Medical University. The healthy donors provided informed consent for the use of blood samples in our research.

### Cell lines and culture

Human renal cell carcinoma cell lines (CAKI-1 and ACHN), 1 human colorectal adenocarcinoma cell line (HT29), human osteosarcoma cell lines (OS2000, KIKU, OS13, HOS, U2OS and HuO9) and 1 human bone malignant fibrous histiocytoma cell line (MFH03) were used. OS2000, KIKU, OS13 and MFH03 were established in our laboratory.^12–15^ The other cell lines were purchased from the Japanese Collection of Research Bioresources Cell Bank (Tokyo, Japan) and from the American Type Culture Collection (Manassas, VA). OS2000, OS13 and MFH03 cells were cultured in Iscove modified Dulbecco’s medium containing 10% FBS, and the others were cultured in Dulbecco’s modified Eagle medium (DMEM) (Sigma-Aldrich, St Louis, MO) containing 10% FBS in a 5% CO_2_ incubator. HT29 transfected with DNAJB8 (HT29-DNAJB8) and HOS transfected with HLA-A*2402 (HOS-A24) were also used.^10,16^ T2-A24 cells lacked the peptide transporter and exogenously expressed HLA-A*24:02.^17^ T2-A24 cells were cultured in RPMI1640 supplemented with G418 used as antigen-presenting cells for exogenously pulsed peptides.

### Biotinylated antigens and peptides

Biotinylated HLA-A*24:02/peptide complex monomers were constructed by Medical & Biological Laboratories, Co., Ltd. (Nagoya, Japan). HLA-A24-restricted peptides DANJB8_143 (AFMEAFSSF), HIV (RYLRDQQLLGI), CMV (QYDPVAALF) and EBV LMP2 (TYGPVFMSL) were used in this study.

### Bio-panning with biotinylated antigen

Bio-panning was performed according to our previous report with some modifications.^18^ Biotinylated HLA–-A24:02/DNAJB8_143 peptide and HLA-A24:02/HIV peptide complexes were used as antigens. The scFv phage-display libraries constructed by our laboratory (Library #1) and provided by Daiichi-Sankyo Co., Ltd. (Library #2) were used for Experiment #1 and Experiment #2, respectively. The phage library (0.25 mL) was mixed with PBS (0.25 mL) containing 4% (w/v) milk in a 1.5-mL tube and incubated with 100 μL of magnetic beads (Dynabeads M-280 Streptavidin, Life Technologies, Carlsbad, CA), which were prewashed with PBS with 0.1% Tween 20 (PBST). The magnetic beads (100 μL) were prewashed and blocked with 2% PBS-M for 1–2 h at room temperature. The phage supernatant was mixed with 0.5 mL of biotinylated HLA-A24:02/HIV peptide complex (1000 nM) and incubated for 60 min (negative panning). After incubation, the phage–antigen mixture was mixed with the magnetic beads for 15 min. The resultant phage supernatant (500 μL) was mixed with 0.5 mL of biotinylated HLA-A24:02/DNAJB8_143 peptide complex (500 nM in the first round and 100 nM in the second and subsequent rounds) and mixed for 60 min (positive panning). After incubation, the phage–antigen mixture was mixed with the magnetic beads, followed by additional incubation for 15 min. Specific phage binders were eluted from the magnetic beads by incubation with 1 mL of 100 mM triethylamine for 7 min. The eluted phage aliquot was immediately neutralised with 100 μL of 1 M Tris-HCl, pH 7.4. The resultant phage aliquot was used for phage rescue.

Phage rescue was performed as follows. Half of the phage aliquot after bio-panning was added to 10 mL of log-phage TG1 or XL1 blue and incubated at 37 °C for 1 h with slow shaking. After incubation, ampicillin (final concentration 100 µg/mL), glucose and helper phage (M13K07 or VCM13) were added and incubated for 60 min. Then, 15 mL of fresh 2xYT containing ampicillin (100 μg/mL) and kanamycin (25 μg/mL) and 1% glucose was added to cultured *E. coli* infected with the phage and helper phage, followed by incubation at 26 °C overnight. Overnight culture of bacteria, including the proliferated phage, was isolated using polyethylene glycol precipitation and used for the next round of bio-panning.

After bio-panning, soluble scFv expression of *E. coli* infected with the phage was induced in a microplate. The phage aliquot after bio-panning (400 μL) was added to 10 mL of log-phage *E. coli* and incubated at 37 °C for 1 h with slow shaking. Following incubation, the *E. coli* aliquot was seeded on a 2xYTAG agar plate and incubated at 37 °C overnight. The next day, 94 clones were picked up and inoculated independently into wells containing 100 μL of 2xYTAG in a 96-well microculture plate. The plate was incubated at 37 °C with shaking for 5 h. After incubation, 20 μL of 2xYTAG with 3 mM isopropyl-β-d(−)-thiogalactopyranoside (IPTG) was added and incubated at 28 °C overnight. Then, 50 μL of the supernatant was harvested and immediately used for ELISA screening according to the previous report.^18^

### Generation of scFv–hIgG and bispecific antibody

scFv cDNA in phagemid vector was subcloned into pFX-hIgG1 for scFv-hIgG1 expression previously constructed by our laboratory.^18^ For soluble expression of scFv-hIgG1, 4 μg of the plasmid was transfected using Lipofectamine 2000 (Life Technologies) into 293 T cells precultured on a 10-cm culture dish in DMEM supplemented with 10% FBS. After 4–5 h, the culture medium was replaced with fresh AIM-V (Life Technologies) without serum. The supernatant was harvested and replaced with fresh AIM-V at 24, 48 and 72 h after transfection. The collected supernatant was passed through a chromatography column with Protein G. The column was washed with 20 mM sodium phosphate (pH 7.0) and eluted by fraction (1 mL per fraction) with a total of 5 mL of 0.1 M glycine (pH 2.7), followed by immediate neutralisation with 1/10 volume of Tris-HCl (pH 9.0). Fractions containing antibodies were assessed by SDS-PAGE with or without DDT to confirm that oxidised scFv–hIgG formed a dimer protein.

The bispecific antibody was constructed as follows: CD3 scFv^19^ linked with a short peptide linker (SGGGGS) and multicloning site (5′-AGTGGCGGCGGAGGATCCAAGAATTCCGCCATGGCAGGTGGCGCGCCAGCGGCCGC-3′) was created by gene synthesis (Integrated DNA Technologies, KK, Tokyo, Japan) and subcloned into pFX-His (CD3-pFX-His). pFx-His was a derivative of pFX-hIgG1 where the hIgG1 constant region was replaced with His tag by our laboratory. scFv cDNA of A10 and B10 was subcloned into CD3 scFv-pFX-His [A10-(CD3×JB8) and B10-(CD3×JB8)]. Soluble expression was performed similar to scFv-hIgG1, followed by Ni-NTA purification using Ni Sepharose 6 Fast Flow (GE Healthcare Japan, Tokyo, Japan).

### Surface plasmon resonance analysis

Surface plasmon resonance analysis was performed using a ProteOn XPR36 (Bio-Rad Laboratories, Inc., Tokyo, Japan) according to the protocol described by Nahshol et al.^20^ Briefly, 1 μg/mL biotinylated monomer (HLA-A*24:02/DNAJB8_143 peptide) in PBS supplemented with 0.005% Tween 20 (PBST) was injected at 30 μL/min at 25 °C and captured on a neutravidin-immobilised NLC sensor tip. Subsequently, serially diluted A10 scFv-hIgG1 and B10 scFv-hIgG1 in PBST was injected at 50 μL/min at 25 °C. All binding sensorgrams were collected and analysed using ProteOn Manager software (Bio-Rad Laboratories, Inc.).

### Immunostaining, flow cytometry and fluorescence microscopy

Before immunostaining, T2-A24 cells (5–10 × 10^5^) were incubated in 200 μL of AIM-V with each peptide at 50 μg/mL (DNAJB8_143, HIV, EBV LMP2 and CMV) on a 96-well round-bottom microculture plate at 26 °C overnight, followed by 2-h incubation at 37 °C. For immunostaining, 5–10 × 10^6^ target cells were seeded in a 96-well microculture plate and incubated with 50–100 μL of 10 μg/mL scFv–hIgG or the supernatant of a hybridoma (C7709A2.6, anti-HLA-A24 mAb)^21^ on ice for 60 min. After two washes, the cells were incubated with 100 μL of anti-human IgG conjugated with PE (1:60 dilution, BioLegend, San Diego, CA) or FITC-conjugated rat anti-mouse IgG antibody (1:200 dilution, KPL, Gaithersburg, MD) for 40 min. The cells were also stained with 50 μL of the bispecific antibody (10 μg/mL) on ice for 60 min followed by anti-His-tag PE (clone AD1.1.10, Abcam). After immunostaining, cells were washed and then fixed with 200 μL of PBS with 0.5% formaldehyde and analysed using FACS Caliber and FACS AriaII (BD Bioscience, San Diego, CA). Fluorescence microscopy assay was performed using BZ-X700 (Keyence Corporation, Osaka, Japan) and Axio Observer.Z1 (Carl-Zeiss, Oberkochen, Germany). T cells and NK cells isolated from PBMC using Pan T Cell Isolation Kit and NK Cell Isolation Kit (Miltenyi Biotec, Gladbach, Germany) were also used.

### Complement-dependent cytotoxicity

Target cells were seeded at 2 × 10^6^ per 100 μL in a 96-well V-bottom plate. Aliquots of 50 μL of antibody (10 μg/mL) were added and incubated for 1 h on ice. After three washes with 0.5% BSA–PBS, cells were incubated with anti-C3b-PE (Clone 3E7/C3b, BioLegend, premixed with human serum) and DAPI (Vector Laboratories Inc., Burlingame, CA), immediately followed by fluorescence microscopy analysis.

### Antibody-dependent cellular cytotoxicity

Target cells were seeded at 1 × 10^5^ per 100 μL in a 96-well V-bottom plate. Aliquots of 50 μL of antibody (10 μg/mL) were added and incubated for 1 h on ice. Isolated T cells were then added (effector:target ratio of 3:1) and co-cultured for 6 h. The frequency of apoptosis of the target cells was assessed by MEBCYTO Apoptosis Kit (Annexin-V-FITC and propidium iodide, MBL International, Woburn, MA) and flow cytometry. In addition, cytotoxicity was assessed using the IMMUNOCYTO Cytotoxicity Detection Kit (MBL International) with carboxyfluorescein succinimidyl ester according to the manufacturer’s protocol.

### Intracellular cytokine staining

Intracellular cytokine staining was performed according to the manufacturer’s protocol. Briefly, T cells were co-cultured with target cells at an effector:target ratio of 3:1. Brefeldin A (BioLegend) was added to the culture after 2 h, followed by further culture for 4 h. Cells were stained with anti-CD45-APC (BioLegend), fixed, permeabilised using the Cytofix/Cytoperm Kit (BD Biosciences) and then stained with anti-IL-2-FITC (clone 5344.111, BD Biosciences), anti-TNFα-PE (clone MAb11, BioLegend) and anti-IFNg-PE-Cy7 (clone 4S.B3, BioLegend). The frequency of cytokine-producing T cells was assessed using flow cytometry.

### Statistical analysis

For comparisons, we used the unpaired *t* test in JMP software (SAS Institute Inc., Tokyo, Japan). Where relevant, figures indicate statistical parameters, including the value of *n*, means ± SD and statistical significance.

## Results

### Isolation of scFv specifically recognising HLA-A24/DNAJB8-derived peptide complex

**Fig. 1:**
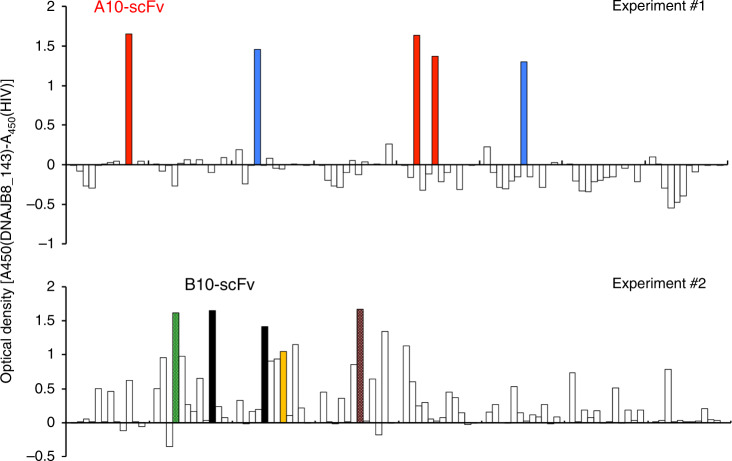
Isolation of scFv clones that recognised the HLA-A24:02/DNAJB8_143 peptide complex. ELISA screening of the specific binders of 94 scFv clones reacting with biotinylated HLA-A*24:02/DNAJB8_143 peptide complex. Experiment #1 and Experiment #2 were performed using Library #1 and Library #2, respectively. The colours indicate highly reactive clones (optical density [A450(DNAJB8_143)-A450(HIV)] > 1.0 and A450(HIV) < 0.1). Colours represent identical CDR3 sequences.

First, we isolated specific scFv clones that recognise the HLA-A24:02/DNAJB8-derived peptide (DNAJB8_143) using naive scFv phage-display libraries. After two independent experiments of three-round bio-panning with biotinylated HLA-A24:02/DNAJB8_143 peptide complex, we isolated several scFv clones that strongly and specifically reacted with the complex (Fig. 1). For further characterisation, we selected A10 scFv and B10 scFv clones, which showed high reactivity with HLA-A24:02/DNAJB8-143 peptide and minimal reactivity with HLA-A24:02/HIV peptide from Experiment #1 and Experiment #2, respectively.

### A10 scFv-hIgG1 and B10 scFv-hIgG1 showed high affinity against the cognate antigen

**Fig. 2:**
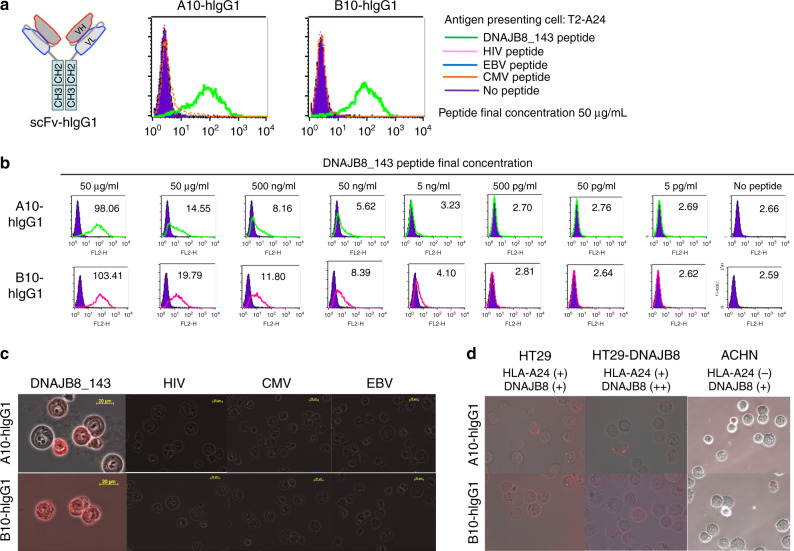
Reactivity of A10-hIgG1 and B10-hIgG1. **a** Reactivity of scFv-hIgG1 to DNAJB8_143 and viral peptides. Each peptide was pulsed at 50 µg/mL. A10-hIgG1 and B10-hIgG1 were used at concentrations of 10 µg/mL. Mean fluorescence intensity (MFI) is indicated. **b** FACS analysis of scFv-hIgG1. T2-A24 cells were pulsed with the indicated peptides and stained with each scFv-hIgG1 at a concentration of 10 µg/mL. Anti-HLA-A24 mAb was used to detect the expression of HLA-A24 molecules. **c**, **d** Fluorescence microscopy of scFv-hIgG1. T2-A24 cells pulsed with the indicated peptides (**c**) and renal cancer cell lines (**d**) were used as targets. Anti-human IgG-PE was used as the second antibody for visualisation. HT29; HLA-A24(+)/DNAJB8(+), HT29/DNAJB8; HLA-A24(+)/DNAJB8(++), ACHN; HLA-A24(−)/DNAJB8(+). Each peptide was pulsed at 50 µg/mL. A10-hIgG1 and B10-hIgG1 were used at concentrations of 10 µg/mL. All experiments were repeated three times.

We constructed A10 and B10 scFv-hIgG1 clones consisting of bivalent scFv and human IgG1 constant region. A10 and B10 scFv-hIgG1 recognised T2-A24 cells pulsed with DNAJB8_143 peptide but not with viral peptides (Figs. 2a, S1). A peptide titration assay using serial dilution of peptides showed that A10-scFv-hIgG1 and B10 scFv-hIgG1 reacted to T2-A24 cells pulsed with the peptide at minimal concentrations of 50 ng/mL and 5 ng/mL, respectively (Fig. 2b). The binding capacity of scFv-hIgG1 clones to DNAJB8_143 peptide presented by HLA-A24 on peptide-pulsed T2-A24 and HLA-A24(+) colorectal carcinoma cell line HT29-DNAJB8 on cell surfaces was also confirmed using fluorescence microscopy (Fig. 2c, d).

Next, the specificity of A10 scFv-hIgG1 and B10 scFv-hIgG1 was assessed using amino acid substitution peptides. The reactivities of the antibodies to amino acid substitution peptide-pulsed T2-A24 cells were determined using flow cytometry. The results showed that position 3 (P3) amino acid, P6 and P7 were critical for recognition (Fig. S2). P2 and P9 might be critical for binding HLA-A24 molecules as anchor amino acids. Considering the importance of the middle part of DNAJB8_143 peptide for specific recognition, A10 scFv and B10 scFv might react specifically with the cognate HLA/peptide complex similar to TCR.

Kinetics analysis of protein interactions between the antibodies and HLA-A24:02/DNAJB8_143 peptide complex was determined by surface plasmon resonance analysis. As shown in Fig. S3, A10 scFv-hIgG1 and B10 scFv-hIgG1 showed strong affinity with the cognate HLA/peptide complex (*K*_D_ = 2.96 × 10^−9^ M and 5.04 × 10^−9^ M, respectively).

### A10 scFv-hIgG1 and B10 scFv-hIgG1 induced complement-dependent cytotoxicity

**Fig. 3:**
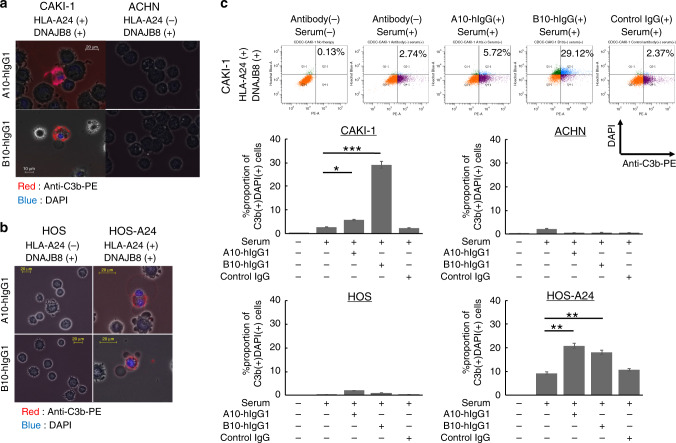
Complement-dependent cytotoxicity (CDC) of A10-hIgG1 and B10-hIgG1. **a** The CDC of scFv-hIgG1 in renal cancer cell line (**a**) and osteosarcoma cell line assessed by fluorescence microscopy. C3b(+)/DAPI(+) cells were indicated as dead cells via CDC. **b** A10-hIgG1 and B10-hIgG1 was used at concentrations of 10 µg/mL. Anti-C3b antibody was used at a concentration of 20 µg/mL. **c** The CDC of scFv-hIgG1 assessed by FACS. The proportion of C3b(+)/DAPI(+) cells is indicated. Data represent the means ± standard error. *p < 0.05, **p < 0.01, ***p < 0.001.

Next, we investigated the capacity of the scFv-hIgG1 clones to induce complement-dependent cytotoxicity (CDC). CDC-induced cells were detected by complement C3b positivity on cell membranes and DAPI positivity in nuclei, which was the result of membrane permeability induced by the classical pathway of CDC. We observed that each of the scFv-hIgG1 clones induced CDC in HLA-A24(+)/DNAJB8(+) renal cell carcinoma cell line CAKI-1 and osteosarcoma cell line HOS-A24 (Fig. 3a, b). The proportion of CDC-induced cells was determined using flow cytometry. Similar to the observation by fluorescence microscopy, we also noted that each scFv-hIgG1 mediated CDC activity on CAKI-1 (5.72% and 29.12%, respectively) and HOS-A24 (20.89% and 18.11%, respectively) by A10 scFv-hIgG1 and B10 scFv-hIgG1 (Fig. 3c).

### Development of bispecific antibody possessing anti-CD3 scFv and anti-HLA-A24/DNAJB8_143 peptide complex scFv

**Fig. 4:**
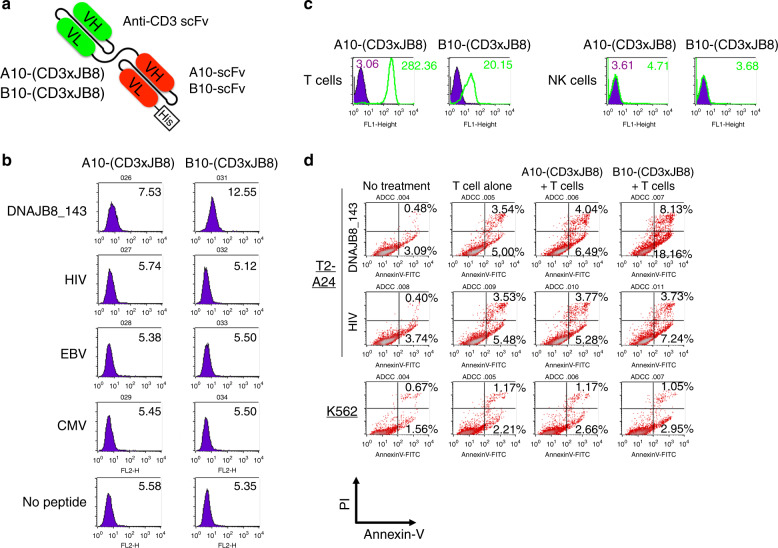

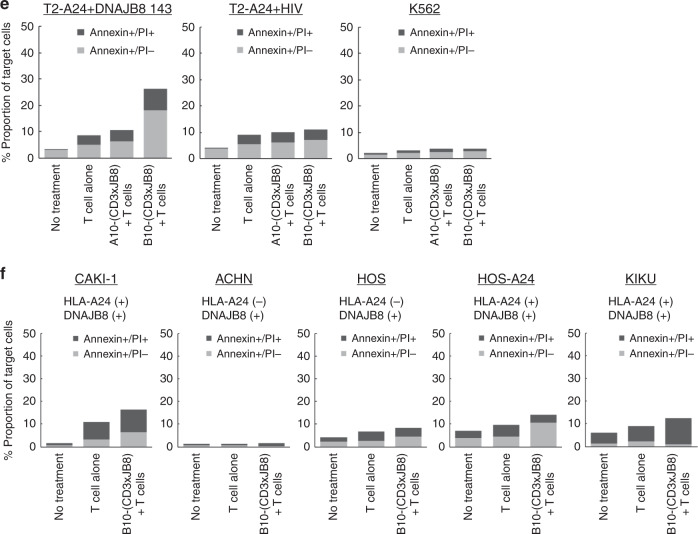
Construction of bispecific antibody reactive to CD3 and HLA-A24/DNAJB8-143 peptide complex. **a** Schematic of the bispecific antibodies, A10-(CD3xJB8) and B10-(CD3xJB8). His tag is located in the C terminus of A10 and B10 scFv. **b** Reactivity of A10-(CD3xJB8) and B10-(CD3xJB8) to peptide-pulsed T2-A24 cells. **c** Reactivity of A10-(CD3xJB8) and B10-(CD3xJB8) to isolated T cells and NK cells. **d**, **e** Antibody-dependent cellular cytotoxicity of A10-(CD3xJB8) and B10-(CD3xJB8) against peptide-pulsed T2-A24 and K562 cells. Annexin-V(+)/propidium iodide (PI)(−) cells indicated early apoptosis, and Annexin-V(+)PI(+) cells indicated late apoptosis. **f** Bispecific antibody-dependent cellular cytotoxicity (BADCC) against renal carcinoma and sarcoma cell lines.

We constructed a bispecific antibody, a combination of anti-CD3 scFv and A10 scFv/B10 scFv with a short GGGS linker (Fig. 4a), which we designated as A10-(CD3xJB8) and B10-(CD3xJB8). We hypothesised that these bispecific antibodies could engage non-specific T cells and HLA-A24(+)/DNAJB8(+) tumour cells and induce cytotoxicity like antibody-dependent cellular cytotoxicity (ADCC). These bispecific antibodies could react with DNAJB8_143 peptide presented by HLA-A24 on cell surfaces and T cells but not NK cells (Figs. 4b, c and S4). However, freshly isolated T-cell-mediated bispecific antibody-dependent cellular cytotoxicity (BADCC) was induced by B10-(CD3xJB8) but barely by A10-(CD3xJB8) (Fig. 4d, e). Therefore, B10-(CD3xJB8) might be suitable for further analysis. We assessed the BADCC capacity of B10-(CD3xJB8) against various cell lines of carcinoma and sarcoma. The results showed that B10-(CD3xJB8) induced BADCC to HLA-A24(+)/DNAJB8(+) cell lines (Figs. 4f and S5). In addition, BADCC capacity was greater when activated T cells were used as the effector cells compared with resting T cells (Fig. S6). Activated T cells via B10-(CD3xJB8) and HLA-A24(+)/DNAJB8(+) target cells produced TNFα predominantly, and IL-2 and IFNγ only slightly (Figs. S7 and S8).

## Discussion

In this study, we (i) isolated specific scFv clones reacting to the HLA-A24-restricted CSC/CIC antigen DNAJB8-derived peptide, and found that (ii) A10 scFv and B10 scFv showed high specificity and strong affinity for the cognate antigen, (iii) bivalent A10 scFv-hIgG1 and B10 scFv-hIgG1 could induce CDC to HLA-24(+)/DNAJB8(+) carcinoma and sarcoma cell lines and (iv) T- cell-engaging bispecific antibody B10-(CD3xJB8) induced T-cell-mediated cellular cytotoxicity against HLA-A24(+)/DNAKB8(+) carcinoma and sarcoma cell lines. These results suggested that such artificial antibodies might be of benefit for antibody-based therapy in patients with carcinoma and sarcoma.

To date, many monoclonal antibodies have been approved and yielded clinical response in haematological malignancies and some solid tumours. However, acquired resistance of tumour cells can occur in monoclonal antibody therapy.^22^ In addition, loss of target antigens has also occurred.^23^ Therefore, new targets and formats of antibodies might be promising for clinical applications. DNAJB8 was previously reported as a CSC/CIC antigen expressed in RCC. Transduction of DNAJB8 increased tumorigenicity, while knockdown of DNAJB8 decreased tumorigenicity in ACHN in vitro and in vivo*.*^10^ Therefore, the antibody targeting DNAJB8-positive tumour cells might be an attractive candidate.

Recently, T-cell-engaging bispecific antibodies have been used particularly in haematological B-cell malignancies.^24^ Blinatumomab (CD3xCD19) showed 43% complete response (CR) and CR with partial haematological recovery (CRh) in patients with Philadelphia-chromosome-negative, primary refractory or relapsed leukaemia.^25^ T cells were activated by CD3 scFv only when they were engaged with CD19-expressing cells via blinatumomab.^24^ Clinical trials of various T-cell-engaging bispecific antibodies, including carcinoembryonic antigen (CEA) × CD3 for gastric cancer, epithelial cell adhesion molecule (EpCAM) × CD3 for epithelial cancer, B-cell maturation antigen (BCMA) × CD3 for multiple myeloma and prostate-specific membrane antigen (PSMA) × CD3 for prostate cancer, are currently being conducted. Most bispecific antibodies target cell surface proteins overexpressed on tumour cells; however, the challenge to develop bispecific antibodies directed to intracellular antigen-derived peptides presented by HLA class I remains. Dao et al. reported the potential efficacy of bispecific antibody directed to WT1-derived peptide in the context of HLA-A2.^26^ Ahmed et al. reported the TCR-like bispecific antibody targeting EBV LMP2A-derived peptide in the context of HLA-A2.^27^ Such bispecific antibodies with TCR specificity might be promising for targeting various tumour-specific intracellular proteins with oncogenic functions. DNAJB8 is a cancer stem-cell/cancer-initiating antigen and not expressed in normal organs, except for testis lacking HLA expression like cancer–testis antigen. Hence, the bispecific antibody targeting DNAJB8, B10-(CD3xJB8), might have therapeutic potential without adverse effects on normal organs.

B10-(CD3xJB8) showed cytotoxicity against HLA-A24(+)/DNAJB8(+) tumour cells; however, the frequencies of IL-2 and IFNγ-secreting cells were lower than those of TNFα. We previously observed that the mRNA expression of TNFα was detectable but not that of IL-2 and IFNγ in naive and stem-cell memory T cells.^28^ Therefore, these observations suggested that B10-(CD3xJB8) might activate T cells close to naive subsets. Further studies using in vivo experiments might be required to prove the efficacy of B10-(CD3xJB8) in the preclinical setting.

In conclusion, we developed scFv clones specific for HLA-A24/cancer stem-like antigen DNAJB8-derived peptide with high specificity and strong affinity. The T-cell-engaging bispecific antibody targeting DNAJB8, B10-(CD3xJB8), might hold promise for antibody-based therapy in patients with carcinoma and bone sarcoma.

## Supplementary information

Supplementary Figure

## Data Availability

The datasets used and analysed during this study are available from the corresponding author.
