# Supplementary material for: Development of an artificial antibody specific for HLA/peptide complex derived from cancer stem-like cell/cancer-initiating cell antigen DNAJB8
Source: Br J Cancer. 2020 Aug 5;123(9):1387–94. doi: 10.1038/s41416-020-1017-1 (PMC7592043; doi:10.1038/s41416-020-1017-1)
Supplement: Supplementary file 1 — Supplementary Figure [file 41416_2020_1017_MOESM1_ESM.pdf]

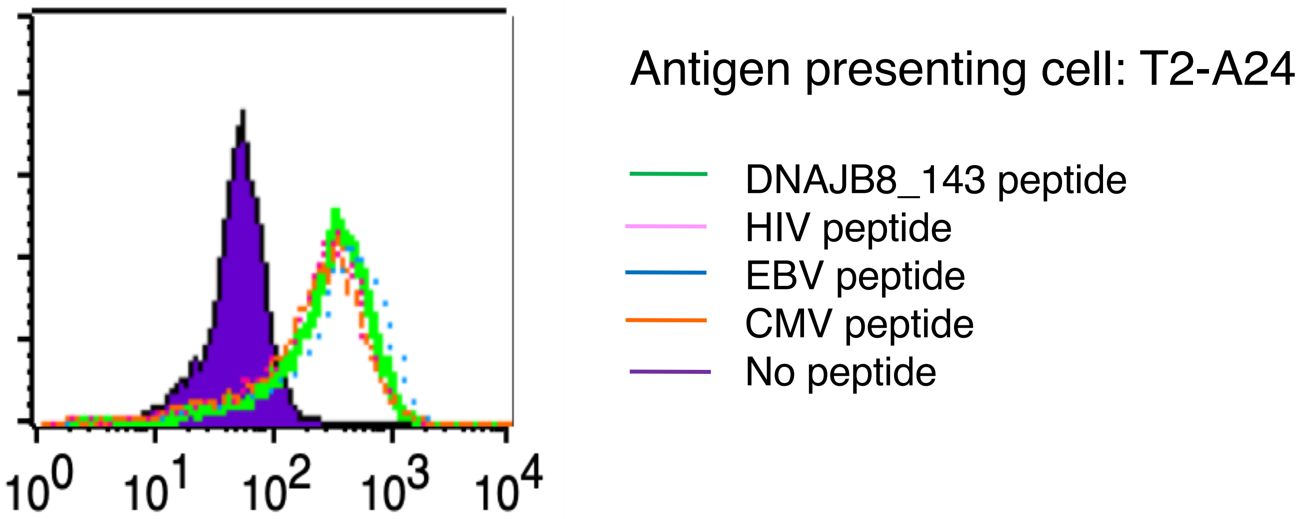

Figure S1. Reactivity of anti-HLA-24 antibody (C7709A2.6) against peptide-pulsed T2-A24 cells. Each peptide was pulsed at 50 μg/mL.

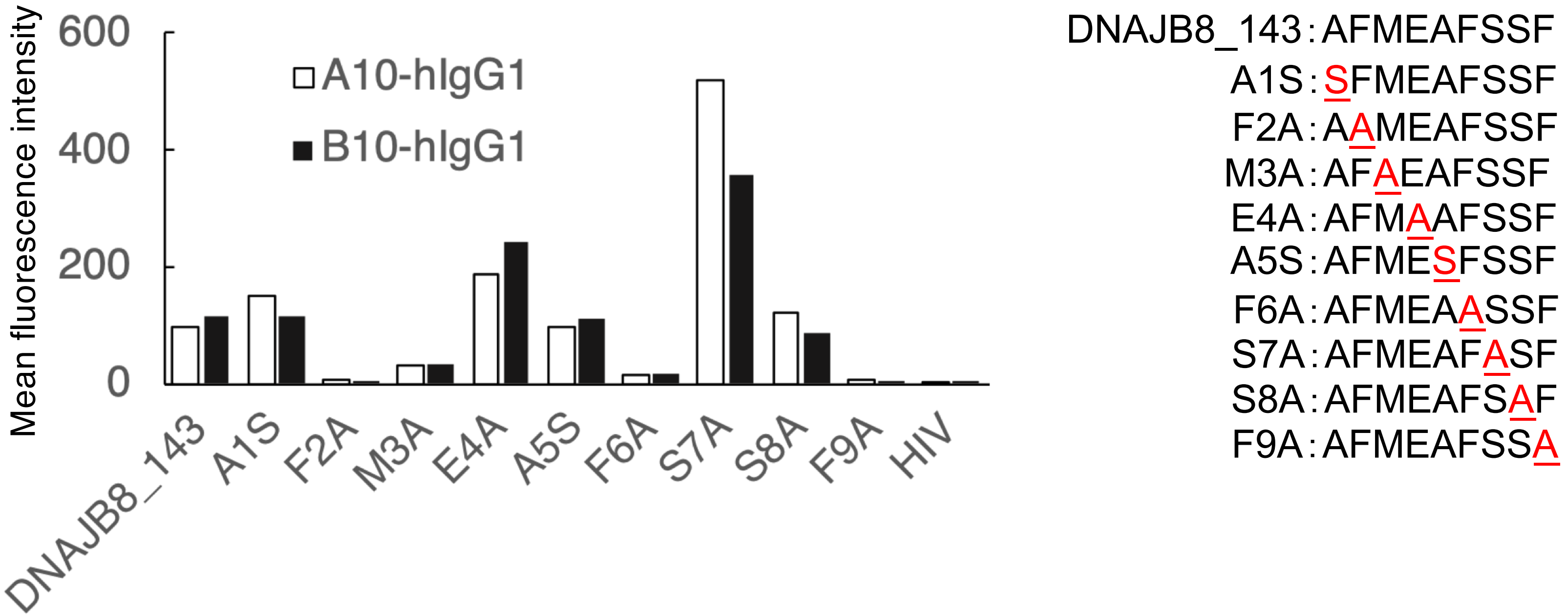

Figure S2. Reactivity of A10-hIgG1 and B10-hIgG1 to anchor substitution peptides presented on T2-A24 cells. Each peptide was pulsed at 50 µg/mL. A10-hIgG1 and B10-hIgG1 were used at concentrations of 10 µg/mL. Mean fluorescence intensity is indicated.

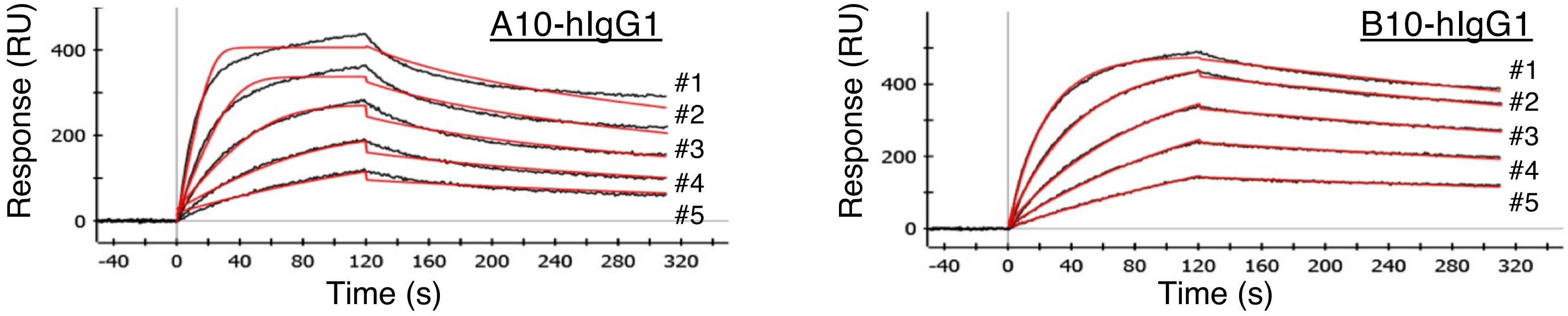

ka=1.52x10^{6} (M^{-1}s^{-1})
Kd=4.50x10^{-3} (s^{-1})
K_D=2.96x10^{-9} (M)

ka=2.21x10^{5} (M^{-1}s^{-1})
Kd=1.11x10^{-3} (s^{-1})
K_D=5.04x10^{-9} (M)

Figure S3. Surface plasmon resonance analysis. Biotinylated HLA-A*24:02/DNAJB8_143 peptide complex was immobilized on the sensor tip as the target. Serially diluted (#1; 10 μg/mL , #2; 5 μg/mL, #3; 2.5 μg/mL, #4; 1.25 μg/mL, #5; 0.625 μg/mL) A10 scFv-hIgG1 and B10 scFv-hIgG1 were used as the analytes.

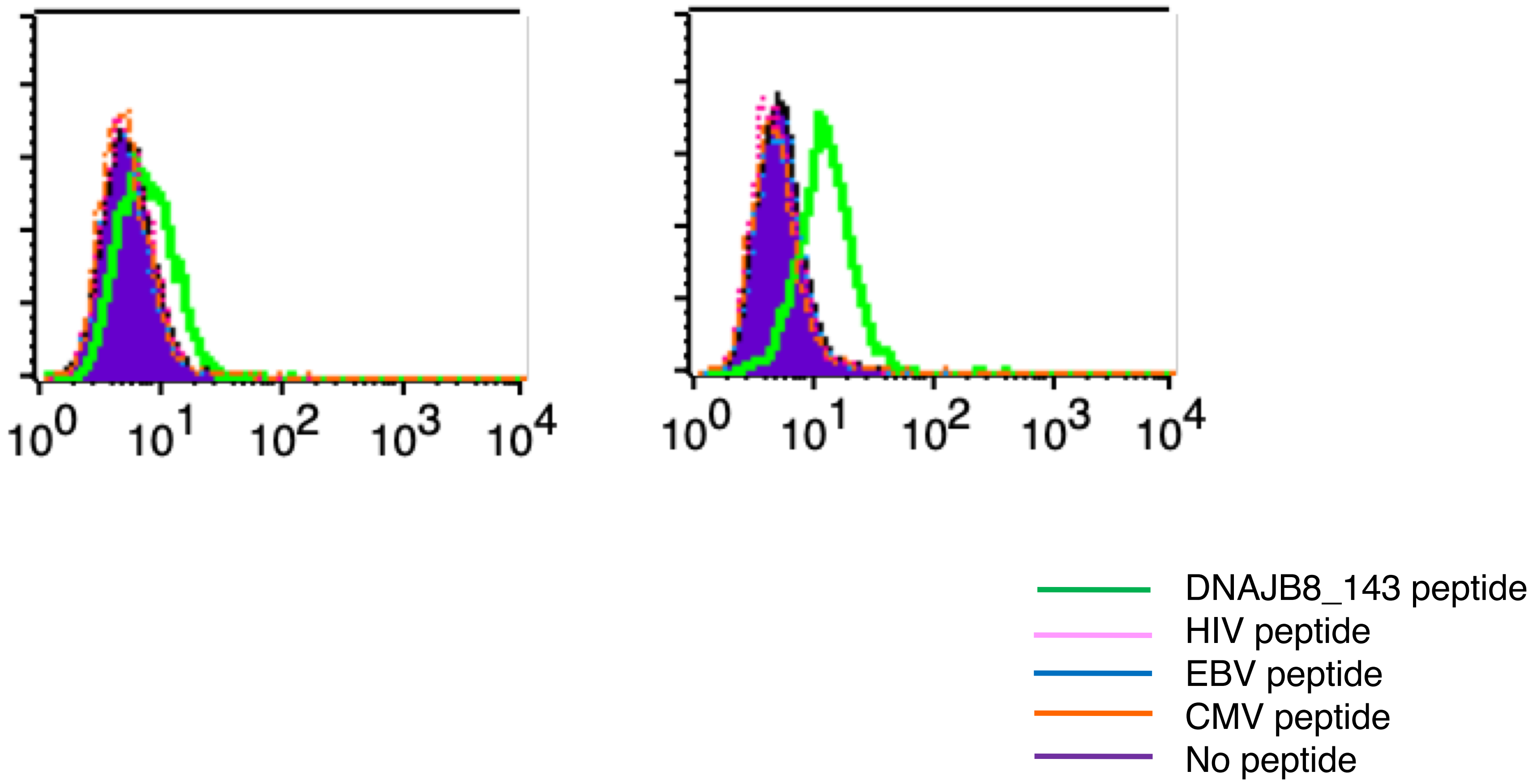

Figure S4. Reactivity of A10-(CD3xJB8) and B10-(CD3xJB8) to T2-A24 cells. T2-A24 cells were pulsed with the indicated peptides.

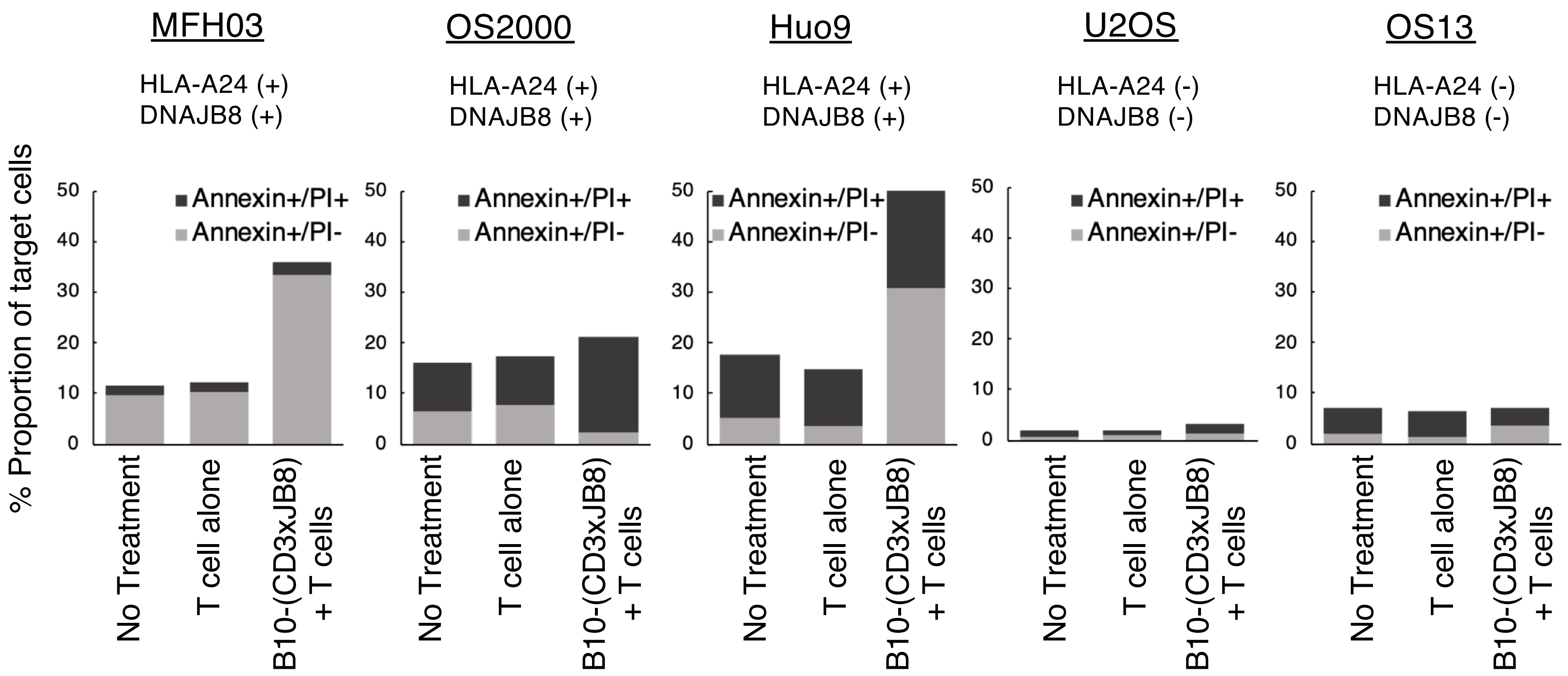

Figure S5. Bispecific antibody-dependent cellular cytotoxicity (BADCC) against renal carcinoma and sarcoma cell lines.

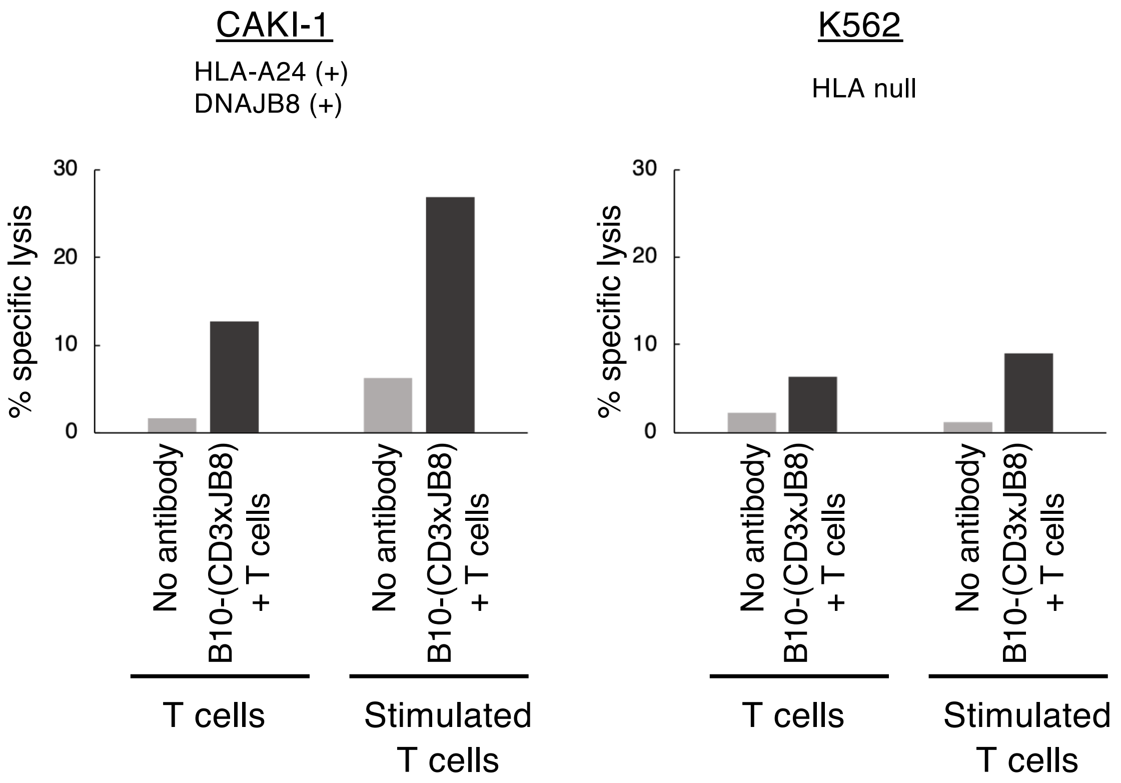

Figure S6. BADCC of B10-(CD3xJB8). Non-stimulated T cells or stimulated T cells with IL-2 (100 U/mL) and soluble OKT3 (50 ng/mL) for 72 h were used as effector cells at an effector:target ratio of 3:1. K562 line as a negative control was used as target cells.

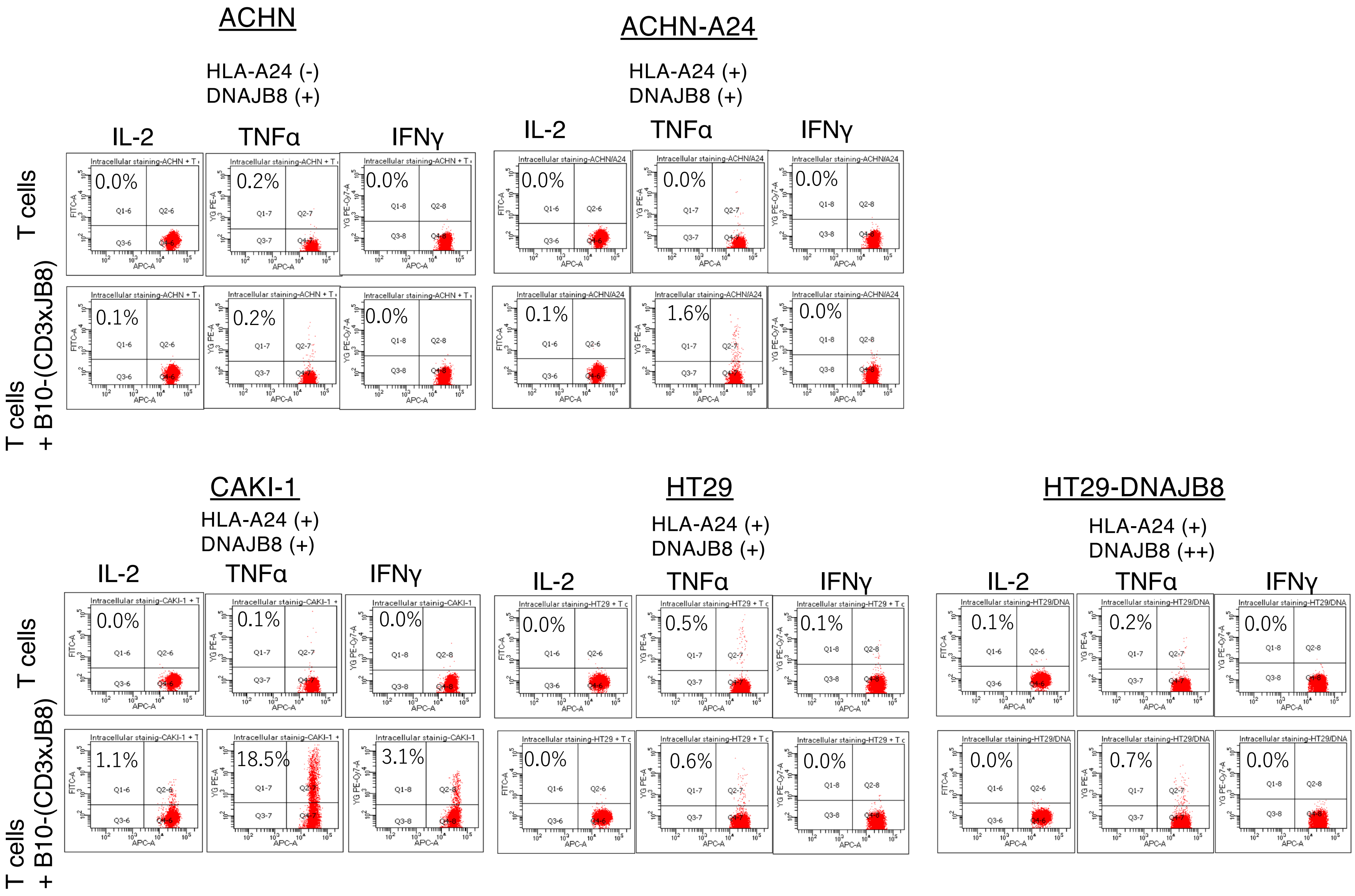

Figure S7. Intracellular cytokine staining. The production of IL-2, TNFα, and IFNγ by T cells was assessed by flow cytometry. Target cells incubated with the antibody were co-cultured with T cells at effector target ratio of 3:1. Brefeldin A was added after 2 h, followed by further culture for 4 h. Renal cell carcinoma and colorectal carcinoma cell lines were used as target cells.

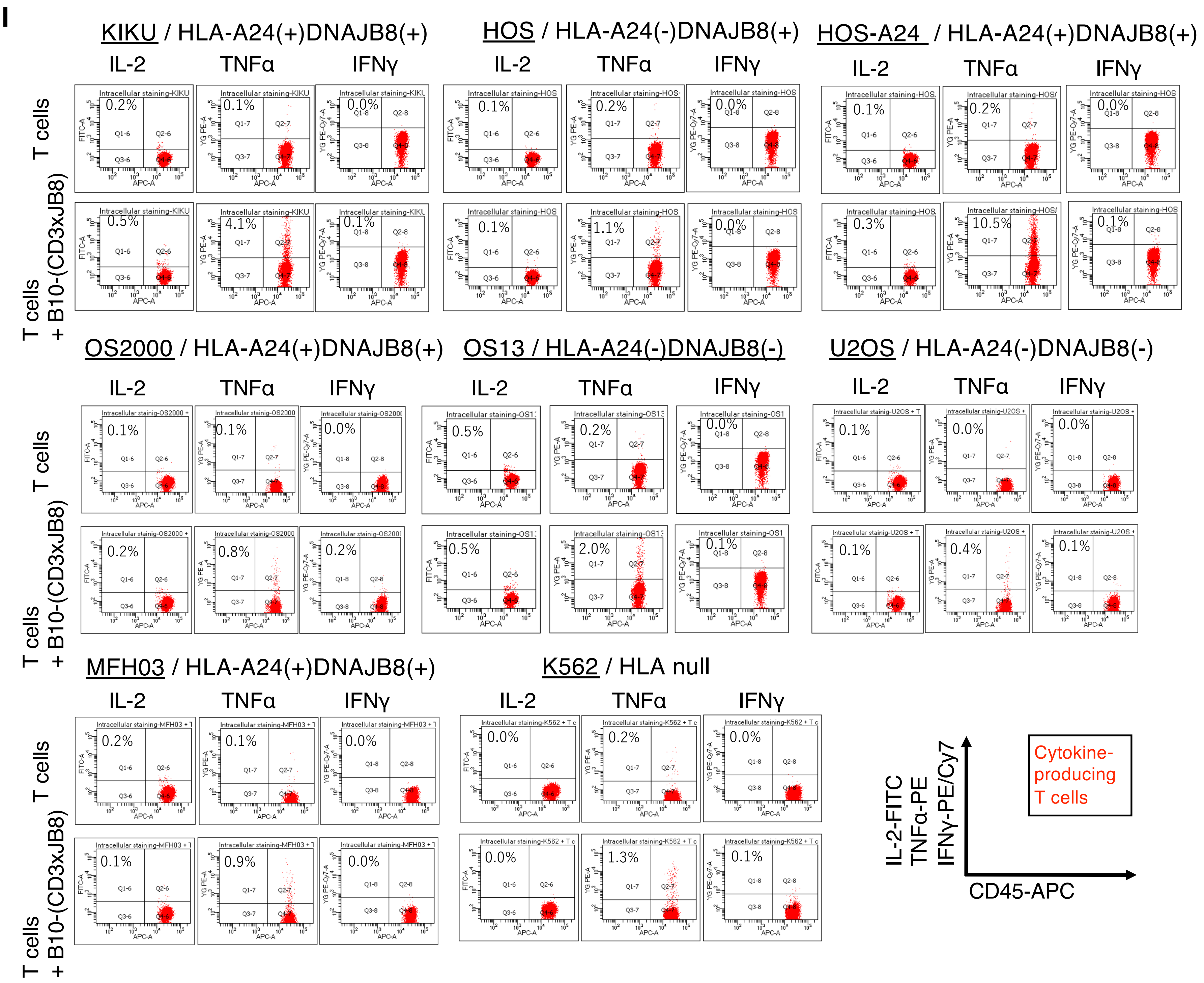
I
KIKU / HLA-A24(+)DNAJB8(+)
HOS / HLA-A24(-)DNAJB8(+)
HOS-A24 / HLA-A24(+)DNAJB8(+)
OS2000 / HLA-A24(+)DNAJB8(+)
OS13 / HLA-A24(-)DNAJB8(-)
U2OS / HLA-A24(-)DNAJB8(-)
MFH03 / HLA-A24(+)DNAJB8(+)
K562 / HLA null
IL-2
TNFα
IFNγ
T cells
T cells + B10-(CD3xJB8)
IL-2-FITC
TNFα-PE
IFNγ-PE/Cy7
CD45-APC
Cytokine-producing T cells

Figure S8. Intracellular cytokine staining. The production of IL-2, TNFα, and IFNγ by T cells was assessed by flow cytometry. Target cells incubated with the antibody were co-cultured with T cells at an effector target ratio of 3:1. Brefeldin A was added after 2 h, followed by further culture for 4 h. Sarcoma cell lines and K562 as a negative control were used as target cells.
